# Use of Early Biomarkers in Neonatal Brain Damage and Sepsis: State of the Art and Future Perspectives

**DOI:** 10.1155/2015/253520

**Published:** 2015-01-18

**Authors:** Iliana Bersani, Cinzia Auriti, Maria Paola Ronchetti, Giusi Prencipe, Diego Gazzolo, Andrea Dotta

**Affiliations:** ^1^Neonatal Intensive Care Unit, Department of Medical and Surgical Neonatology, Bambino Gesù Children's Hospital, IRCCS, Piazza Sant'Onofrio 4, Rome, Italy; ^2^Department of Rheumatology, Bambino Gesù Children's Hospital, IRCCS, Piazza Sant'Onofrio 4, Rome, Italy; ^3^Department of Maternal, Fetal and Neonatal Medicine, C. Arrigo Children's Hospital, Alessandria, Italy

## Abstract

The identification of early noninvasive biochemical markers of disease is a crucial issue of the current scientific research, particularly during the first period of life, since it could provide useful and precocious diagnostic information when clinical and radiological signs are still silent. The ideal biomarker should be practical and sensitive in the precocious identification of at risk patients. An earlier diagnosis may lead to a larger therapeutic window and improve neonatal outcome. Brain damage and sepsis are common causes of severe morbidity with poor outcome and mortality during the perinatal period. A large number of potential biomarkers, including neuroproteins, calcium binding proteins, enzymes, oxidative stress markers, vasoactive agents, and inflammatory mediators, have been so far investigated. The aim of the present review was to provide a brief overview of some of the more commonly investigated biomarkers used in case of neonatal brain damage and sepsis.

## 1. Introduction

### 1.1. Biomarkers: An Overview

The use of noninvasive laboratory biomarkers has become a key element in clinical practice throughout the last decades. The research of new biological markers enabling a precocious identification of neonates at risk of neonatal diseases, allowing a close monitoring of the disease and providing information about prognosis, represents a strategic objective of several current researches. According to the US National Institutes of Health (NIH), specific definitions have been assigned to the terms “biomarker,” “clinical endpoint,” and “surrogate endpoint.” A “biomarker” is a characteristic which is objectively measured and evaluated as an indicator of physiologic biological processes, pathogenic processes, or pharmacologic responses to a therapeutic treatment. A “clinical endpoint” is a characteristic or variable reflecting patients' feeling, functions, or survival. A “surrogate endpoint” is a biomarker which is intended to be a substitute for a clinical endpoint [1]. The International Program on Chemical Safety, led by the World Health Organization (WHO) together with the United Nations and the International Labor Organization, defined a biomarker as “any substance, structure, or process that can be measured in the body or its products and influences or predicts the incidence of outcome or disease” [2]. The development of a biological marker starts with the discovery and identification of a new biomarker, is followed by a close evaluation of its accuracy, and, thereafter, evaluates the impact of the marker on clinical outcomes [3]. To date, the identification of reliable biomarkers of diseases may have many potential applications either in research or in clinical medicine [4].

The clinical and radiological signs of a large number of neonatal diseases develop belatedly in a wide percentage of patients. Therefore, the identification of early biochemical markers of disease is a crucial issue of the current scientific research, since it could provide useful and precocious diagnostic information when clinical and radiological signs are still silent. The ideal biomarker should be practical and sensitive in the precocious identification of at risk patients: this would allow an early identification of the population at risk, enabling preventive or therapeutic strategies.

In order to be reliably used in the perinatal medicine, biomarkers should be studied well in the pediatric population, measured by means of worldwide available commercial kits, characterized by adequate reproducibility, comparable with ranges of normality available also for term and preterm neonates, and investigated in different biological fluids (blood, urine, CSF, saliva, milk, and amniotic fluid) and implying only low testing-related discomfort for the neonates.

Although the present narrative review does not have the robustness of a systematic review or a meta-analysis of diagnostic test studies, its aim was to provide a brief overview of the current status of knowledge about some of the most promising biomarkers which could play a role in neonatal clinical practice. Since neonatal brain damage and sepsis often complicate neonatal clinical course, we focused our attention on these two potentially severe conditions. Therefore, in the present review we provide information about the most widely used biomarkers of neonatal brain damage and sepsis.

## 2. Biochemical Markers of Perinatal Brain Damage

The perinatal period is a crucial time point for proper brain development. The occurrence of clinical complications such as intrauterine growth retardation (IUGR), maternal diabetes, hypotension, cerebral ischemia, and reperfusion during this life period may lead to an amplified release of vasoactive molecules, inducing exaggerated cell death and tissue damage. Perinatal asphyxia occurs in about 0.2–0.4% of term neonates, leading to lethal hypoxic ischemic encephalopathy (HIE) in 20% of cases and to permanent neurodevelopment impairment in 25% of cases. Intraventricular hemorrhage (IVH) represents a possible consequence of perinatal asphyxia affecting both term and preterm neonates, although the risk of IVH is inversely related to gestational age and birth weight. Unfortunately, the early postinsult period is in most cases clinically and radiologically silent despite an underlying, already ongoing, biochemical brain damage. Therefore, the detection of biomarkers allowing the early identification of infants at risk of brain damage represents a major goal of current scientific investigations, since it could amplify the therapeutic window and, consecutively, improve neonatal outcome [5]. Another important goal is to identify the biomarker providing the most accurate information about the quantitative extension of brain damage and representing a reliable marker for the evaluation of therapy effectiveness [6]. A number of possible early markers of brain damage have been investigated throughout the last decades, such as the S100B, adrenomedullin (AM), erythropoietin (EPO), activin A, neuron-specific enolase (NSE), oxidative stress markers (OS), glial fibrillary acidic protein (G-FAP), and creatine kinase BB (CK-BB) (Table 1).

### 2.1. S100B

S100B is an acidic calcium-binding peptide of the S100 family of proteins specific for the nervous tissue. It is mostly expressed by glial cells and in neuronal subpopulations [7, 8] and exerts paracrine and autocrine effects on neurons and glia. A dual effect of S100B on cell function has been described: low S100B levels improve cell function while high concentrations lead to impaired cell activity by increasing nitric oxide production [9].

S100B concentrations in the cerebrospinal fluid (CSF) were a reliable biomarker among neonates with perinatal asphyxia in the evaluation of brain lesion extent and correlated with the neurologic impairment at one year of age as well as with death before one year [10, 11].

Blood S100 half-life is 1 hour and a mostly renal excretion has been reported. Blood S100B concentrations have been investigated as expression of an ongoing brain damage. Cord S100B concentrations were higher in case of perinatal asphyxia and HIE [12]. In particular, S100B blood peak was recorded 6 hours after birth and was followed by a progressive decrease at 24 hours [12]. High concentrations of this protein have been reported in both term [12, 13] and preterm [14] neonates with HIE already 48–72 hours before the development of any clinical, laboratory, or ultrasonographic signs of IVH. This peculiar property of the S100B may enable prompt medical interventions before the occurrence of irreversible brain damage. Furthermore, a correlation between both increased cerebrovascular resistances and IVH extent and S100B blood levels has been highlighted [13, 14]. High levels of this protein were found among neonates with IUGR as a consequence of the so-called brain sparing effect [15]. Even maternal S100B concentrations were higher in case of neonates affected by IUGR and IVH [16]. Blood S100B measurement has been also investigated as a tool for the identification of infants subjected to extracorporeal membrane oxygenation (ECMO) at risk of intracranial haemorrhage when imaging assessment and clinical symptoms of haemorrhage might still be silent [17, 18].

Considering its renal excretion, S100B levels have been investigated also in the urine. Urine S100B concentrations resulted to be age-dependent [19]. Increased levels of urine S100B were highlighted among preterm neonates developing IVH before the development of any clinical biochemical or radiological signs [20]. Moreover, higher concentrations of urine S100B have been found in term neonates with perinatal asphyxia showing neurologic impairment on follow-up [21]. As a whole, these data suggest that S100B may help in the early determination of prognosis after brain injury.

### 2.2. Adrenomedullin (AM)

AM is a vasodilatory hypotensive peptide which was isolated for the first time from human pheochromocytoma [22]. Human AM is a 52-amino-acid peptide which is synthesized as part of a larger precursor molecule termed preproadrenomedullin. The AM gene, mapped on chromosome 11, is expressed in a wide range of tissues [23]. AM plasma half-life is 22 hours and the lung seems to be a major site of human AM clearance [24]. A number of biological effects of AM have been highlighted throughout the years [25] but AM's main effect is represented by an increased production of cyclic adenosine monophosphate [22]. A possible role for AM in cardiovascular adaptation after birth and as biomarker of heart failure has been described [26, 27]. Blood AM levels have been also investigated among cardiopathic children undergoing cardiopulmonary bypass [28]. An upregulation of the AM gene has been also reported in case of inflammation [29] and its greatest increase was documented in case of septic shock [30]. Furthermore, AM is highly expressed in the brain and has shown neuropeptide characteristics. Increased AM concentrations have been documented in case of hypoxia [31, 32], and a possible protective role in this case has been postulated. Moreover, AM concentrations increased in case of perinatal asphyxia among neonates who developed IVH: this seems to mirror the cerebral vascular regulation loss after perinatal asphyxia and has been suggested as possible biomarker for the identification of neonates at risk of neurologic impairment [33].

### 2.3. Erythropoietin (EPO)

EPO and its receptor are expressed in astrocytes, neurons, and endothelial brain cells [34]. A prospective pilot cohort study investigating cord blood concentrations of EPO in preterm infants found that EPO levels were higher among those neonates who would have developed IVH, and this result was confirmed after correction for GA [35]. Since EPO production increases after fetal hypoxia [36, 37], high EPO concentrations in cord blood may reflect a fetal hypoxic status predisposing to IVH.

### 2.4. Activin A

Activin A is a member of the TGF-beta superfamily and is made up by two *βα* subunits [38]. Activin A promotes neuronal differentiation [39], its receptors are highly expressed in neuronal cells, and neuronal activity upregulates activin mRNA expression [40]. It was originally purified from gonadal fluids as a protein enhancing the pituitary follicle stimulating hormone release. However, a wide range of biological activities of the activin proteins have been described, such as mesoderm induction, neural cell differentiation, bone remodeling, hematopoiesis, and roles in reproductive physiology. Enhanced expression of activin A seems to represent a common response to acute neuronal damage of various origins, including perinatal asphyxia [41]. Studies investigating the role of activin A as a biomarker of brain damage found increased cord concentrations among preterm neonates developing IVH within 72 hours of life [42]. Increased CSF levels were detected in term neonates with perinatal asphyxia and the highest concentrations were found among neonates with the most severe HIE [43]. Also urine concentrations of activin A measured at birth were higher among neonates with moderate or severe HIE than among neonates with no or mild HIE [44].

Besides its possible role as a biomarker for brain injury, activin A has also been investigated in patients with heart failure, who showed significantly higher serum concentrations and increased gene expression of activin A compared with healthy control subjects. Therefore, some authors suggested an involvement of activin A in the pathogenesis of heart failure [45].

### 2.5. Neuron-Specific Enolase (NSE)

The NSE is a 78 kD gamma-homodimer representing the main enolase-isoenzyme detectable in neuronal and neuroendocrine tissues. NSE is detectable in both CSF and blood (after impairment of the blood-brain barrier, stroke, or brain injury). The main limitation to a widespread use of NSE is that, for accurate NSE determinations, serum samples must be free from haemolysis [46]. Although very few data exist concerning NSE use as biomarker of brain damage in the perinatal period, some authors found that NSE increased in asphyxiated neonates [47, 48] and that high serum NSE levels measured during hypothermia were associated with neuroradiographic and clinical evidence of brain injury among encephalopathic neonates [48].

### 2.6. Oxidative Stress Markers (OS)

The term “oxidative stress” describes an unbalance between prooxidant and antioxidant factors potentially leading to cellular and tissue damage, and the OS are known to be involved in the pathogenesis of several fetal and neonatal diseases [49, 50]. In particular, the immature antioxidant system characterizing preterm infants may be insufficient to counteract OS damaging effects. Some authors recently described a correlation between OS degree and the severity of damage in the perinatal period. However, OS reference curves in all human fluids are still lacking, and studies investigating the possible correlations between OS and the true extent of brain injury are still lacking.

### 2.7. Glial Fibrillary Acidic Protein (G-FAP)

The G-FAP is a monomeric filament protein localized predominantly in astroglial cells. G-FAP is released as a consequence of brain damage and astrogliosis. Increased levels have been detected after stroke occurrence [51]. G-FAP progressively increases according to the postmenstrual age in both term and preterm neonates, although preterm infants with abnormal neurologic outcome show higher levels compared to healthy term neonates [52]. Moreover, serum G-FAP concentrations among neonates suffering from HIE seem to be predictive of brain injury on MRI [53]. However, only few data still exist concerning the use of G-FAP as biomarker for the long term neurologic outcome and further investigations are required.

### 2.8. Creatine Kinase BB (CK-BB)

The CK-BB isoenzyme is a protein detectable in both neurons and astrocytes. Some studies suggested its use as biomarker of brain damage during the neonatal period, especially if considered in combination with the S100B protein [12]. However, nonunanimous results have been achieved by different studies about the use of CK-BB as predictive marker for adverse outcome after perinatal asphyxia. Some authors demonstrated a correlation between increased CK-BB activity at 6–12 h of life and the following neurologic outcome [54], while other authors reported only a weak correlation between its levels at 4 hours of life and the neurologic impairment [55]. Moreover, no significant correlation between high CK-BB levels and impaired neurologic outcome could be demonstrated by a large retrospective study [56]. A further confounding factor is that CK-BB might arise from noncerebral tissues, since it is also expressed in placenta, gastrointestinal tract, kidneys, and lungs [57].

## 3. Biochemical Markers of Sepsis

Neonatal sepsis represents a major complication of the neonatal period. Its beginning may be slow and characterized by subtle, late, and unspecific symptoms in some cases, but fulminant sepsis with quick deterioration of neonates' clinical status may also occur. In both circumstances, an increased risk of neonatal death is reported among either preterm or term neonates. Since the early detection of neonates at risk of sepsis could enlarge the therapeutic window and improve neonatal outcome, several markers of sepsis have been investigated throughout the last years as possible tools for an early identification [58–60]. The most studied biomarkers include C-reactive protein, procalcitonin, mannose-binding lectin (MBL), serum amyloid A (SAA), proinflammatory cytokines (IL-6, IL-8), adrenomedullin, lipopolysaccharide-binding protein (LBP), triggering receptor expressed on myeloid cells (sTREM), and urokinase plasminogen activator receptor (suPAR) (Table 2). Unfortunately, no unanimous data about the most appropriate biomarker for neonatal sepsis have been achieved yet and the best diagnostic strategies in this field are still widely discussed.

### 3.1. C-Reactive Protein (CRP)

The CRP is a globulin that forms a precipitate when combined with the C-polysaccharide of* Streptococcus pneumoniae*. It is an acute phase protein released by the liver. IL-6 and other proinflammatory cytokines such as IL-1 influence the hepatic production of CRP. Due to the delayed induction of hepatic synthesis its production in neonates increases at 4–6 hours after stimulation and peaks at 48 hours. Although CRP is widely used in neonatal clinical practice, its accuracy in the differentiation between infectious and noninfectious diseases is not exact, since inflammatory conditions not of microbial origin may also lead to increased CRP levels, limiting the use of single values [60].

The sensitivity of CRP is known to be the lowest during the early stages of infection [61], while its diagnostic accuracy improves by the performance of serial CRP determinations and by its combination with earlier markers such as interleukins or procalcitonin. Serial CRP determinations seem to be useful also for the monitoring of treatment response [62]. Lower baseline CRP concentrations and a lower CRP response to infection have been suggested in case of preterm compared to term neonates. Therefore, some authors suggest that the currently most used cut-off value of 10 mg/L is not perfectly suitable for preterm neonates. Moreover, since during the first three days of life a physiologic tendency to increased CRP rise has been highlighted, mostly secondary to the stress of delivery [63], not only gestational age but also postnatal age may affect CRP concentrations [62].

### 3.2. Procalcitonin

Procalcitonin, a propeptide of calcitonin, is an acute phase reactant which increases at 4 hours and peaks already at 6–8 hours after the stimulus [58, 64]. This means that, compared to CRP, procalcitonin has the advantage of a more rapid increase. Neonates with proven or clinically diagnosed bacterial infection show increased levels of procalcitonin [65]. Moreover, a recent meta-analysis suggested that procalcitonin was characterized by higher accuracy than the CRP for the diagnosis of late-onset sepsis [66]. As for CRP, physiologic variations of procalcitonin concentrations have been reported among uninfected neonates, with a peak on days 1-2 of life, followed by a progressive decrease [63]. These physiologic changes imply that the cut-off values should consider postnatal age. In a recent study from our group we recommended a procalcitonin cut-off value of >2.4 ng/mL as the most accurate level for differentiation of sepsis in neonates, regardless of gestational age, with a sensitivity of 62% and a specificity of 84% [67]. This cut-off value exceeds the physiologic increased procalcitonin median value of 2,38 ng/mL, observed in uninfected infants in the second day of life (data unpublished). Moreover, also the occurrence of respiratory distress syndrome or haemodynamic failure affects procalcitonin levels during the first 10 days of life among preterm and term neonates [68]. These data, together with the changeable basal levels of procalcitonin, limited its use as single laboratory marker of sepsis. However, a combination of procalcitonin with further biomarkers such CRP seems to improve the accuracy in the detection of infected neonates. Furthermore, procalcitonin seems to be a reliable marker also for the monitoring of antibiotic effectiveness [65].

### 3.3. Mannose-Binding Lectin (MBL)

The MBL is a member of the collectin family which is produced by the liver as an acute phase protein. MBL binds to a wide range of pathogens including bacteria, viruses, and fungi [69, 70]. MBL activates macrophages, enhances phagocytosis, and cooperates in complement activation [70, 71]. Low serum MBL levels increase the risk of infections, especially if associated with other conditions such as immune deficiencies of various origins. Low serum MBL concentrations have been reported also among neonates with sepsis, suggesting a possible role of MBL as biomarker for the early identification of neonates at risk of infection [72–75]. A prospective observational study performed at our institution, including 365 critically ill neonates, found that median MBL serum concentrations were significantly lower among infected than among uninfected neonates. Moreover, low MBL levels on admission increased the risk of infection, independently on GA and invasive procedures. Nevertheless, MBL levels on admission and the peak levels during infection were not associated with death [75]. It should be underlined that a significant interindividual variability of serum MBL concentrations has been reported. More specifically, particularly low MBL levels have been detected among preterm neonates [76] and a genetically determined MBL deficiency has been described [76, 77]. The MBL monomer is encoded by the MBL-2 gene placed on chromosome 10, and functional MBL is made up by a polymer of several MBL monomers. Single nucleotide polymorphisms (SNPs) of the MBL-2 gene interfere with the assembly of the protein and/or the protein expression. The decrease of the functional circulating MBL affects the effectiveness of the immune response to infections. The abovementioned study carried out at our institution highlighted no significant differences in the MBL-2 genotype variants between infected and uninfected neonates [75]. These results were in agreement with a previous study including preterm neonates admitted to the NICU, which found no relationship between MBL genotype and the risk of nosocomial sepsis or pneumonia [78].

### 3.4. Cytokines

In case of sepsis an activation of the inflammatory cascade leads to increased production of a large number of proinflammatory cytokines. Their significance as early biomarkers of infection is greater than the one of the acute phase proteins, since cytokines production precedes and induces the synthesis of acute phase reactants. IL-1, IL-6, IL-8, and TNF*α* are the cytokines which have been more widely investigated as biomarkers for neonatal sepsis. All of them showed increased serum concentrations among septic neonates [79–81]. Compared to CRP, IL-6 increases more precociously but, due to its short half-life, its levels fall to the baseline value within 24 hours. Therefore, IL-6 may represent a precocious marker of neonatal sepsis but in most circumstances clinicians may arrive too late for the detection of increased levels [79, 82]. This means that increased CRP levels in the absence of high IL-6 concentrations may indicate an inflammatory process begun at least 24–48 hours before. As for IL-6, comparable increasing times have been also reported for TNF*α* [79]. IL-1*β* also increases in case of sepsis but neonatal blood levels among preterm infants are much lower than those detected among adult septic patients, maybe because of inadequate secretion by the immune cells during this life period. It should be underlined that these cytokines may increase also in case of inflammation not being of infectious origin. In contrast, some authors suggested a discriminative role of IL-8 between infections and uninfective inflammatory processes [83].

### 3.5. Adrenomedullin (AM)

Some of the main biologic properties of AM have already been described above in the text. Interestingly, increased levels of serum AM have been reported in case of sepsis and septic shock [30, 84]. Unfortunately, only poor data still exist about its use among neonates.

### 3.6. Soluble Triggering Receptor Expressed on Myeloid Cells-1 (sTREM-1)

The TREM-1 is a 30 kDa glycoprotein of the immunoglobulin superfamily which is selectively expressed in neutrophils and monocytes/macrophages [85]. It is still unclear if TREM directly binds to bacterial products, as the toll-like receptors, or not. Regardless of the nature of TREM-1 ligand, the TREM-1 is able to initiate and amplify the inflammatory cascade. During sepsis, activated phagocytes release the sTREM-1 [86], which has been suggested as a reliable biomarker for bacterial infections [87]. However, sTREM-1 may be also detectable in case of noninfectious inflammatory diseases. Increased concentrations of sTREM-1 have been recorded in case of neonatal sepsis [88], although the true role of this molecule as an early biomarker of sepsis has been only poorly investigated in the perinatal period.

### 3.7. Lipopolysaccharide-Binding Protein (LBP)

The LBP is a 50 kDa protein of the acute phase which is mainly synthesized in the liver. It binds with high affinity to the lipopolysaccharide in the plasma, transfers the LPS to the membrane-bound CD14 or to its soluble form of CD14, and affects the inflammatory response against microbial stimuli. Serum LBP is constitutively detectable but increased levels have been reported among adults with Gram-negative, Gram-positive, and fungal infection [89, 90]. Increased LBP concentrations have been described also among term and preterm neonates affected by early-onset sepsis [91–94]. Nevertheless, despite the large number of studies describing LBP activity in vitro and in animal models, very few data still exist concerning its clinical reliability as biomarker of infection among neonates and children.

### 3.8. Serum Amyloid A (SAA)

The term SAA describes a family of 12 to 14 kDa polymorphic apolipoproteins which are mainly produced by the liver as acute phase proteins. A rapid and marked increase of SAA occurs within 8–24 h after the onset of sepsis. High levels of SAA have been reported also among septic neonates [95–98]. Some authors suggested a more rapid increase after the beginning of sepsis compared to CRP [98], and this could allow a more precocious identification of septic neonates. Its use as biomarker of neonatal sepsis seems to be enforced by its combination with other inflammatory markers such as CRP and procalcitonin [95]. SAA seems to have a role also during neonatal follow-up to evaluate the response to treatment [95].

### 3.9. Soluble Urokinase Plasminogen Activator Receptor (suPAR)

The uPAR is a protein embedded in the cell membranes of some immune cells including neutrophils, macrophages, and lymphocytes. suPAR is involved in several immunologic functions including chemotaxis, cell migration, cell adhesion, immune activation, and tissue remodelling [99]. Its soluble counterpart (suPAR) has been reported to increase in case of infection. Increased serum levels of suPAR seem to predict bacteremia in adult patients with SIRS [100–103]. Moreover, increased suPAR levels seem to have a better prognostic value than procalcitonin and CRP [104]. However, very poor data exists investigating the role of suPAR in the neonatal period.

## 4. Conclusions

The early identification of neonatal diseases represents a major issue of current research. The use of biochemical markers of disease, detectable when the clinical and radiological signs are still silent, may enable either preventive or therapeutic strategies of treatment. To date, no unanimous opinions exist about the gold standard biomarkers concerning two common diseases of the perinatal period such as brain damage and NEC.

Among the laboratory markers studied for the early diagnosis of brain damage, S100B seems to be the most reliable one.

Concerning neonatal sepsis, despite the promising results for some diagnostic biomarkers, current knowledge has not identified yet a single marker able to diagnose alone 100% of infected cases. A combination of biomarkers may provide more reliable information for early diagnosis.

Further investigations are required to achieve definitive results and optimize the diagnostic strategies in neonatal clinical practice.

## Figures and Tables

**Table 1:** Biomarkers of brain injury in the neonatal period: reference curves availability and assays.

Biomarkers of brain injury	Reference curves in neonates	Assay
S100B	Y	Chemiluminescence/ELISA
AM	NA	ELISA
EPO	Incomplete	Chemiluminescence/ELISA
Activin A	Incomplete	ELISA
NSE	NA	ELISA
OS	Incomplete	ELISA/HPLC
G-FAP	NA	ELISA
CK-BB	Incomplete	Electrophoresis

AM: adrenomedullin; EPO: erythropoietin; NSE: neuron-specific enolase; OS: oxidative stress markers; G-FAP: glial fibrillary acidic protein; CK-BB: creatine kinase BB; Y: yes; NA: not available.

**Table 2:** Biomarkers of sepsis in the neonatal period: reference curves availability and assays.

Biomarkers of sepsis	Reference curves in neonates	Assay
CRP	Y	Immunoturbidimetric/immunonephelometric assay
Procalcitonin	Y	Immunoluminometric assay
MBL	Incomplete	ELISA
IL-6	Y	Flow Cytometry
AM	NA	ELISA
sTREM	NA	double antibody sandwich ELISA
LBP	NA	ELISA
SAA	Incomplete	Immunonephelometric assay
suPAR	NA	ELISA

CRP: C-reactive protein; MBL: mannose-binding lectin; IL: interleukin; AM: adrenomedullin; sTREM: triggering receptor expressed on myeloid cells-1; LBP: lipopolysaccharide-binding protein; SAA: serum amyloid A; suPAR: urokinase plasminogen activator receptor; Y: yes; NA: not available.

## Abbreviations

AM: Adrenomedullin
CK-BB: Creatine kinase BB
CSF: Cerebrospinal fluid
CRP: C-reactive protein
EPO: Erythropoietin
G-FAP: Glial fibrillary acidic protein
HIE: Hypoxic ischemic encephalopathy
IL: Interleukin
IUGR: Intrauterine growth retardation
IVH: Intraventricular haemorrhage
LBP: Lipopolysaccharide-binding protein
MBL: Mannose-binding lectin
NSE: Neuron-specific enolase
OS: Oxidative stress markers
SAA: Serum amyloid A
TREM-1: Triggering receptor expressed on myeloid cells-1
suPAR: Urokinase plasminogen activator receptor.
